# Analysis of the Causes on Poor Clinical Efficacy of Kyphoplasty Performed in Unilateral Transpedicular Puncture for the Treatment of Senile Osteoporotic Vertebral Compression Fractures

**DOI:** 10.1038/s41598-018-37727-9

**Published:** 2019-02-06

**Authors:** Hao Yin, Xuejun He, Huijun Yi, Zhiguo Luo, Jianmin Chen

**Affiliations:** Department of Orthopaedic Surgery, Hunan Provincial People’s Hospital, Changsha, People’s Republic of China

## Abstract

This study intends to analyze the causes on poor clinical efficacy of kyphoplasty performed in unilateral transpedicular puncture for the treatment of senile osteoporotic vertebral compression fractures. A retrospective study was conducted on a consecutive series of 70 patients who had underwent kyphoplasty performed in unilateral transpedicular puncture for the treatment of senile osteoporotic vertebral compression fractures between March 2016 to March 2017. These patients were compared for clinical data to investigate the causes on poor clinical efficacy of kyphoplasty performed in unilateral transpedicular puncture for the treatment of senile osteoporotic vertebral compression fractures. Comparison result of the indices between these patients showed that the differences in body weight, fracture type and bone cement dispersion were statistically significant. Logistic multivariate regression analysis showed body weight (OR = 0.892, p = 0.042), fracture type 2 (OR = 0.089, p = 0.020) and bone cement dispersion (OR = 4.773, p = 0.025) are risk factors for poor clinical efficacy. The results of corresponding analysis on VAS (Visual Analogue Scale), vertebral height and Cobb angle in patients with poor clinical efficacy showed that there is a correlation between them. We believe that patients’ weight, dispersion degree of bone cement and fracture type of injured vertebra are the risk factors of kyphoplasty with poor clinical efficacy.

## Introduction

With the aging of the population, vertebral compression fractures have become the most common complication of osteoporosis^1^. Studies have reported that the mortality rate of vertebral compression fractures is significantly higher than that of other diseases associated with osteoporosis, seriously affecting the life quality of middle-aged and elderly people^2,3^. Vertebral compression fractures, as the most common type of fracture in middle-aged and elderly people, are often accompanied by the clinical manifestations such as kyphosis deformity and lumbago and back pain^4^. At present, conservative and surgical treatments are used clinically, but related studies have reported poor efficacy of conservative treatment and even the possibility of aggravating vertebral collapse^5^. In recent years, with the continuous development of spinal minimally invasive technique, kyphoplasty has been widely used in vertebral compression fractures. At present, unilateral or bilateral transpedicular puncture is mainly used in percutaneous kyphoplasty. Studies have shown that both unilateral and bilateral transpedicular punctures have therapeutic effects on pain and can quickly improve patients’ quality of life^6–8^. But it is necessary to repeat the operation twice in bilateral transpedicular puncture, use two sets of operating instruments, and increase operation time and radiation exposure time, which also increase patients’ economic burden. Therefore, more and more scholars tend to use unilateral transpedicular puncture approach^9–11^. However, in clinical use of unilateral transpedicular percutaneous kyphoplasty for the treatment of osteoporotic vertebral compression fractures, we found that some patients had no significant relief of postoperative pain symptoms or still had residual partial pain. Why did this happen? We are very confused about it, and there were few reports about the causes of this poor clinical efficacy. The purpose of this study was to treat osteoporotic vertebral compression fractures by unilateral transpedicular percutaneous kyphoplasty and to conduct clinical analysis for the patients with poor clinical efficacy, in order to explore related mechanism resulting in this poor clinical efficacy.

## Results

### Analysis on the number of the patients

70 patients with osteoporotic thoracic and lumbar fractures were followed up for 8–24 months, with an average of 12.3 months.

### Surgical situation

The operations were successfully completed in the 70 patients. The observation results on pain relief within 24 hours after surgery showed that there were no complications such as spinal nerve injury and pulmonary embolism.

### The leakage and complications of bone cement

According to the postoperative X-ray images, it was found that there were 2 patients with bone cement leakage who didn’t cause clinical symptoms, and there were 2 patients who had non-adjacent other vertebral fractures at the end of the follow-up.

### The comparison of the basic conditions

There were 10 patients with the VAS score ≥5 points on the first day after surgery was assessed as the group with poor clinical efficacy. There were 60 patients with the VAS score <5 points on the first day after surgery was assessed as the group with good clinical efficacy. Comparison result of the indices between the two groups showed that the differences in body weight (Table 1), fracture type (Table 2), postoperative VAS (Table 3) and bone cement dispersion (Table 2) were statistically significant between the two groups. From the comparison of the basic conditions, it could be seen that the patients with poor efficacy were higher than those with good efficacy in body weight, the proportion of fracture type 2 and bone cement dispersion.

**Table 1:** Comparison of basic situation of patients with different curative effects.

Index	Curative Effect		Statistical value	P
	Poor (n = 10)	Good (n = 60)		
Age	71.30 ± 13.14	72.87 ± 8.36	t = −0.502	0.617
Male/Female	5/5	15/45	χ^2^ = 2.625	0.105
Weight	65.50 ± 10.74	57.97 ± 7.98	t = 2.626	0.011
Duration of disease	3 (1~22)	2 (1~7)	z = −0.737	0.461
Causes of trauma history	3 (30.0%)	20 (33.3%)	χ^2^ = 0.043	0.835

**Table 2:** Comparison of patients with different curative effects before and after operation.

Index		Curative Effect		Statistical value	P
		Poor (n = 10)	Good (n = 60)		
Segments	1	7 (70.0%)	50 (83.3%)	z = −0.919	0.358
	2	3 (30.0%)	8 (13.3%)		
	3	0 (0.0%)	2 (3.3%)		
AO Classification	1	2 (20.0%)	41 (68.3%)	χ^2^ = 8.451	0.004
	2	8 (80.0%)	19 (31.7%)		
Bone cement leakage		1 (10.0%)	1 (1.7%)	—	0.267
Staging of fracture time	1	7 (70.0%)	48 (80.0%)	z = 0.704	0.482
	2	2 (20.0%)	8 (13.3%)		
	3	1 (10.0%)	4 (6.7%)		
Bone cement dispersion	1	3 (30.0%)	2 (3.3%)	z = −3.143	0.002
	2	5 (50.0%)	18 (30.0%)		
	3	2 (20.0%)	40 (66.7%)

**Table 3:** Comparison of postoperative follow-up of patients with different curative effects.

Index	Curative Effect		Statistical value	P
	Poor (n = 10)	Good (n = 60)		
Pre-operative VAS	7.5 (7~8)	7 (6~8)	z = −0.651	0.515
Post-operative VAS	7 (6~7)	3 (2~3)	z = −4.433	0.000
One year post-operative VAS	2 (1.75~3.25)	2 (2~3)	z = −0.462	0.644
Pre-operative vertebral height	18.53 ± 6.02	16.65 ± 5.84	t = 0.939	0.351
Post-operative vertebral height	21.62 ± 5.42	20.10 ± 5.62	t = 0.795	0.429
One year post-operative vertebral height	17.70 ± 4.56	17.29 ± 4.92	t = 0.245	0.807
Pre-operative Cobb	22.2 (3.7~30.35)	15.55 (7.23~25.78)	z = −0.730	0.465
Post-operative Cobb	12.35 (3.05~18.88)	11.65 (5.43~22.4)	z = −0.386	0.699
One year post-operative Cobb	18.8 (6.45~20.4)	15.35 (8~24.38)	z = −0.201	0.840

### The risk factors for poor clinical efficacy

Logistic multivariate regression analysis was performed on the three factors with statistical significance in the univariate analysis. The result (Table 4) showed body weight (OR = 0.892, *p* = 0.042), fracture type 2 (OR = 0.089, *p* = 0.020) and bone cement dispersion (OR = 4.773, *p* = 0.025) are risk factors for poor efficacy.

**Table 4:** Multiple stepwise regression analysis of logistic related factors.

Index	B	S.E,	Wals	df	Sig.	Exp (B)	EXP (B) 95% C.I.	
							Lower Limit	Upper Limit
Weight	−0.115	0.057	4.116	1.000	0.042	0.892	0.798	0.996
AO Classification	−2.420	1.043	5.383	1.000	0.020	0.089	0.012	0.687
Bone cement dispersion	1.563	0.697	5.032	1.000	0.025	4.773	1.218	18.698
Constant	9.104	4.944	3.390	1.000	0.066	8988.482		

### The results of corresponding analysis on VAS, vertebral height and Cobb angle

**Figure 1:**
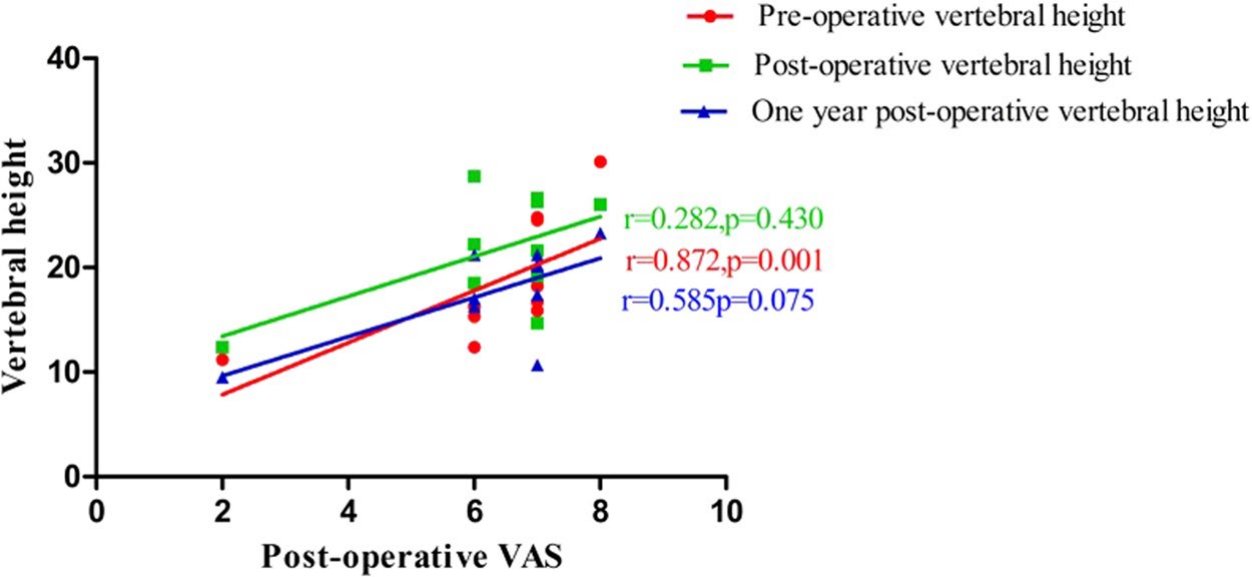
The relationship between vertebral height and Post-operative Vas in patients with poor efficacy. Postoperative VAS was positively correlated with preoperative vertebral height (r = 0.872, *p* < 0.01).

**Figure 2:**
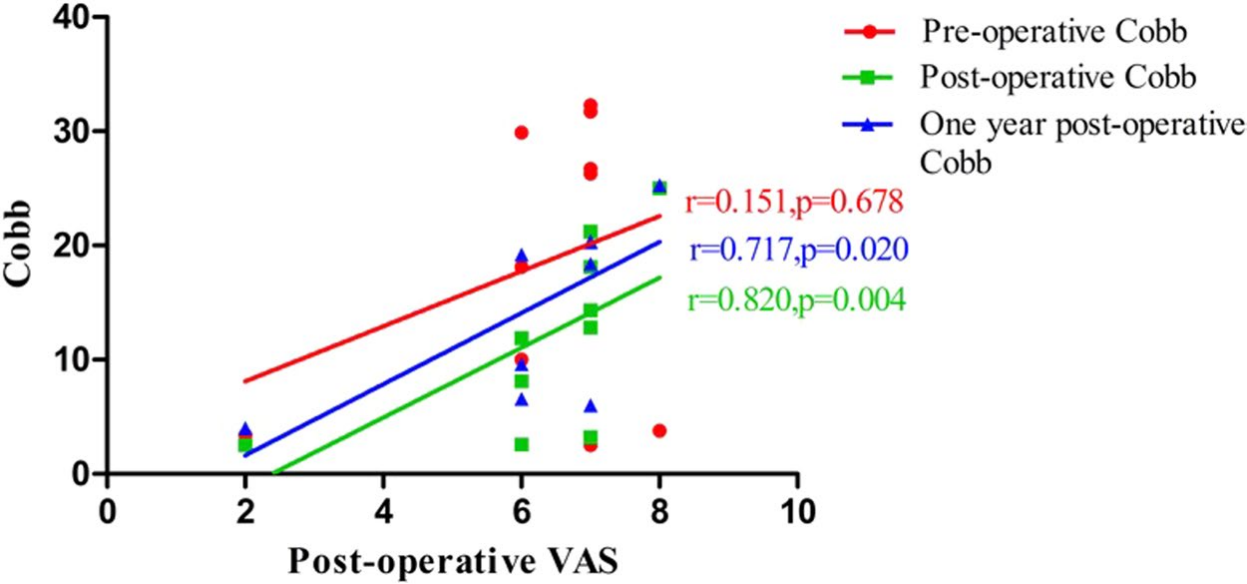
The relationship between Cobb and Post-operative Vas in patients with poor efficacy. Postoperative VAS was positively correlated with postoperative Cobb angle (r = 0.820, *p* < 0.01) and the Cobb angle in postoperative 1 year (r = 0.717, *p* < 0.01).

**Figure 3:**
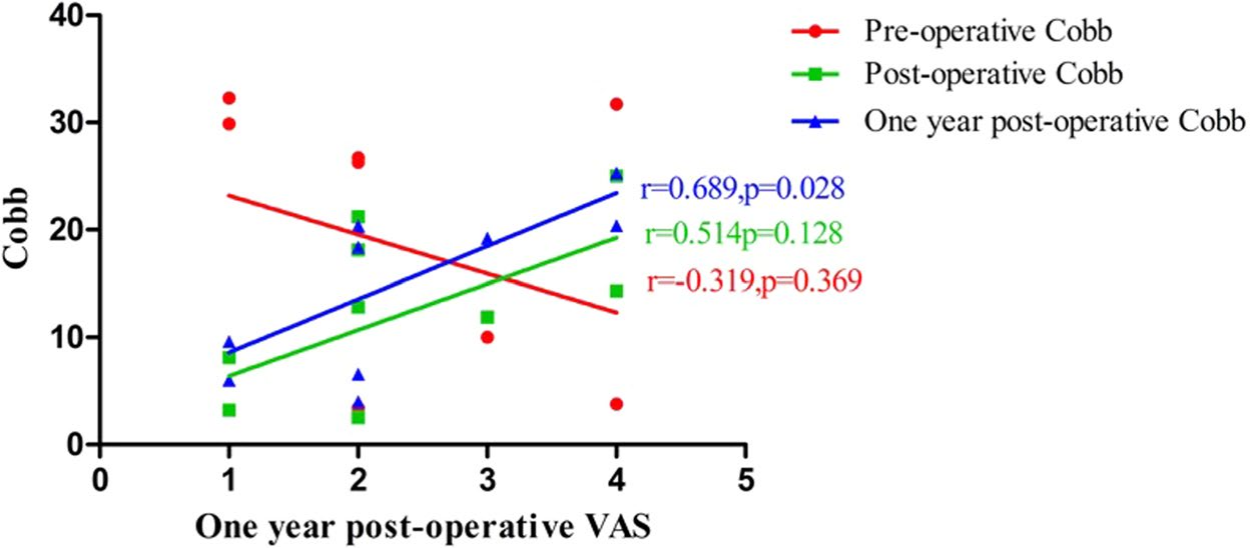
The relationship between Cobb and one year Post-operative Vas in patients with poor efficacy. There was a positive correlation between the VAS value and the Cobb angle in 1 year after surgery (r = 0.689, *p* < 0.01).

The results (Table 5) of corresponding analysis on VAS, vertebral height and Cobb angle in patients with poor efficacy showed that postoperative VAS was positively correlated with preoperative vertebral height (r = 0.872, *p* < 0.01) (Fig. 1), and was also positively correlated with postoperative Cobb angle (r = 0.820, *p* < 0.01) (Fig. 2) and the Cobb angle in postoperative 1 year (r = 0.717, *p* < 0.01) (Fig. 2). It was suggested that vertebral height, postoperative Cobb angle and the Cobb angle in postoperative 1 year as well as the postoperative VAS had an increasing trend. There was also a positive correlation between the VAS value and the Cobb angle in 1 year after surgery (r = 0.689, *p* < 0.01) (Fig. 3). In addition, the correlations between preoperative vertebral height and postoperative Cobb angle (r = 0.770, *p* < 0.01), between preoperative vertebral height and the vertebral height in postoperative 1 year (r = 0.851, *p* < 0.01), and between postoperative Cobb angle and the Cobb angle in postoperative 1 year, were also very significant.

**Table 5:** Correlation analysis results of VAS, Vertebral height and Cobb in patients with poor efficacy (Spearman).

	Pre-operative VAS	Post-operative VAS	One year post-operative VAS	Pre-operative vertebral height	Post-operative vertebral height	One year post-operative vertebral height	Pre-operative Cobb	Post-operative Cobb	One year post-operative Cobb
Pre-operative VAS	1.000	0.266	−0.399	0.142	0.039	0.013	0.582	0.175	0.045
Post-operative VAS		1.000	0.324	0.872^**^	0.282	0.585	0.151	0.820^**^	0.717^*^
One year post-operative VAS			1.000	0.130	0.189	0.447	−0.319	0.514	0.689^*^
Pre-operative vertebral height				1.000	0.236	0.383	−0.139	0.770^**^	0.602
Post-operative vertebral height					1.000	0.851^**^	−0.055	0.248	0.231
One year post-operative vertebral height						1.000	0.067	0.505	0.543
Pre-operative Cobb							1.000	−0.200	−0.122
Post-operative Cobb								1.000	0.936^**^
One year post-operative Cobb									1.000

Note: * indicates P < 0.05, ** indicates P < 0.01.

## Discussion

Osteoporosis is a common disease that strikes the elderly, with an incidence rate of about 6.6%^12^. It is characterized by decreased bone mass, decreased bone strength and increased risk of fracture. Spinal fractures caused by osteoporosis have become an important cause of disability and death, seriously affecting the quality of life of the elderly^13,14^. The curative effect of kyphoplasty for the treatment of osteoporotic vertebral compression fractures is definite, through which pain can be quickly relieved, bed rest time reduced, normal living conditions restored in shortest time possible, quality of life improved, and incidence rate of complications in elderly patients caused by factors such as long-term bed rest and decreased activity reduced. As has been reported^15–17^ in a large number of literatures at home and abroad, for the elderly patients, kyphoplasty is the best practice for the treatment of osteoporotic vertebral compression fractures, featuring advantages of minimal invasion, shorter operation time, faster symptom relief, effective pain relief, reduction of complications, etc. Ample reports^18,19^ pointed out that when treating osteoporotic thoracolumbar vertebral compression fractures with percutaneous kyphoplasty (PKP), both unilateral transpedicular puncture and bilateral transpedicular puncture can achieve satisfactory results. However, it is still a subject of much debate regarding the choice for unilateral transpedicular puncture or bilateral transpedicular puncture during clinical treatment. Some scholars believe that bilateral transpedicular puncture can produce better long-term effect for it can evenly strengthen fractured vertebral bodies, with lower rate of bone cement leakage. While unilateral transpedicular puncture, seeking higher success rate of puncture and better dispersion effect, often requires a larger leaning angle during puncture, which may increase the risk of spinal cord and nerve injury and the leakage of bone cement^20–23^. Other reports^24–27^ suggested that unilateral transpedicular puncture is more advantageous as it requires less time, causes less trauma, and has less chance of being exposed to X-rays for patients and doctors during surgery without causing the increase of bone cement leakage. Some scholars argue that there is uneven bone cement dispersion using unilateral transpedicular puncture, making the enhancement on the left and right of vertebral bodies become asymmetrical and serving as a risk factor for vertebral refracture or adjacent segmental fracture^28,29^. However, bilateral transpedicular puncture can avoid asymmetry of such enhancement caused by bone cement dispersion difference and achieve holistic enhancement of vertebral bodies^30^. Satisfactory curative effect is achieved in treating patients with osteoporotic thoracolumbar vertebral fractures using unilateral transpedicular puncture PKP in clinical practice, with most patients receiving good results and still a few patients whose postoperative pain symptoms not being effectively relieved. This makes us confused. What is the cause of this phenomenon? We will make a preliminary discussion through this research.

The results from Tables 1 and 2 show that, the difference concerning body weight, fracture type and degree of bone cement dispersion between the two groups is statistically significant. According to the comparison result of basic situation, patients with poor efficacy have higher body weight than those with good efficacy, so is the proportion of fracture type 2 (there are injuries on the upper and lower endplates of injured vertebrae) and degree of bone cement dispersion. The results in Table 3 suggest that comparative changes regarding vertebral height and Cobb angle before surgery, after surgery and 1 year after surgery respectively are not statistically significant. The results in Table 4 indicate that body weight, fracture type 2 and degree of bone cement dispersion are risk factors for unsatisfactory efficacy. By reviewing the literature^31,32^, it is revealed that kyphoplasty is an effective minimally invasive surgical procedure for the treatment of vertebral compression fractures. Its main principle is to inject filler into vertebral body through cannula, and to correct deformity and relieve pain by destructing vertebral sensory nerve endings and strengthening stability of vertebral body after bone cement solidifies through heat release. Zhao^33^ *et al*. believe that, in percutaneous kyphoplasty, bone cement with various dispersion levels can effectively alleviate pain caused by vertebral compression fractures. After vertebra plasty, the effect of bone cement dispersion level on spinal dysfunction index suggest statistical significance, and such grading method is of clinical guiding significance for the evaluation of early spinal function after percutaneous vertebroplasty (PVP). Fan^34^ displays through experiment that bone cement filling is mostly confined to the side of vertebral puncture by unilateral vertebral pedicle puncture, resulting in mechanical imbalance on both sides of vertebral body. Bone cement filling of the unilateral puncture group is mostly limited to the lateral side of the responsible vertebral body, and bone cement filling of the bilateral transpedicular puncture group is mostly distributed in the central area of the responsible vertebral body. The difference in the distribution of bone cement in vertebral body caused by different surgical methods may be the reason why the stiffness and strength of the three groups of spinal units are different. By improving the puncture method, the bone cement can be diffusely distributed in the center of the vertebral body through unilateral vertebral pedicle perfusion in order to fill the injured vertebrae, thereby making the mechanical transmission of the entire spinal column unit more balanced. We believe that, for patients with damages on bilateral endplates, unstable injured vertebrae and insufficient bone cement dispersion are the reason for unsatisfactory postoperative efficacy of PKP. Patients with unsatisfactory postoperative outcome may be correlated with incomplete stabilization of injured vertebrae and incomplete destruction of nerve endings in the vertebral body. In these patients, it is found through follow-up visits that patients with unsatisfactory curative effect using unilateral transpedicular puncture lose no Cobb angle and vertebral height after surgery.

For seeking better curative effect and less complications using unilateral transpedicular puncture, Wu^35^ *et al*. consider that dispersion volume obtained by injecting 3.5 mL bone cement may be comparable to that by injecting 4.5 ml. Qi^36^ *et al*. believe that, specific analysis on the choice for unilateral PVP treatment or bilateral PVP treatment for patients with osteoporotic vertebral compression fractures should be made based on specific circumstances. For example, if the collapse degree of the diseased vertebra is not serious and the fracture is limited to one side of the vertebral body, puncture should be made on the lesion side; if the collapse degree of the diseased vertebra is severe, puncture should be made on the opposite side of the lesion; if the vertebral pedicle on one side is destroyed, puncture should be made on the side with relatively complete vertebral pedicle; if the diseased vertebral body collapses evenly, puncture should be made on bilateral sides. In the actual situation, if it is unable to determine whether bilateral or unilateral transpedicular puncture should be performed, the puncture should be conducted on the side with more odds of success and based on the distribution of bone cement on this side, whether or not conducting contralateral puncture can be determined. Tu^37^ *et al*. believe that unilateral or bilateral transpedicular puncture should be selected as per MRI images features of patients’ vertebrae in an attempt to shorten operation time as much as possible and reduce surgical risk of elderly patients. For vertebral body with obvious fissure images or vacuum signs inside, unilateral transpedicular puncture can obtain satisfactory dispersion effect; for vertebral body in which high signals are shown locally, bilateral transpedicular puncture can be performed to achieve better dispersion effect and lower leakage rate of bone cement.

According to the results in Table 5, it could be seen that VAS, height of injured vertebra and the change in Cobb angle are correlated in patients demonstrating poor curative effect. Figures 1 and 2 show that postoperative VAS is positively correlated with preoperative vertebral height, postoperative Cobb angle and Cobb angle 1 year after surgery, suggesting that the higher preoperative vertebral height, postoperative Cobb angle and Cobb angle 1 year after surgery, the higher postoperative VAS. Figure 3 show that VAS value 1 year after surgery is positively correlated with cobb value 1 year after surgery. In addition, the correlation between preoperative vertebral height and postoperative cobb, postoperative vertebral height and vertebral height 1 year after surgery, postoperative cobb and cobb 1 year after surgery is also significant. The mechanism of this situation is currently unclear. It may be related to the change in spinal curvature after PKP surgery. Jin^38^ *et al*. believe that: It can be concluded that proper correction of kyphosis deformity and restoration of vertebral height can restore normal spinal physiological curvature; however, higher correction angle and correction height are not necessarily better, and further biomechanical studies and clinical studies are needed for determining the degree of recovery involving kyphosis angle and vertebral height. Some scholars hold that vertebroplasty increases the risk of recurrent fractures of adjacent vertebral bodies^39^. As for the related factors of adjacent vertebral fractures, studies find that the stiffness of the vertebral body rises and the degeneration of intervertebral disc accelerates after injecting bone cement into the vertebral body when performing PVP or PKP, especially when the bone cement leaks into the intervertebral disc where the risk of adjacent vertebral fractures increases^40,41^; however, it is found in a prospective multicenter randomized controlled study led by Klazen^42^ that intervertebral disc leakage is not correlated with the incidence rate of adjacent vertebral refracture. Clinical studies reveal that refractures of adjacent vertebral bodies are related to bone mineral density index and kyphosis angle, and the recovery degree of kyphosis angle is positively correlated with the incidence rate of refractures of adjacent vertebral bodies, stressing that excessive recovery of vertebral height and kyphosis angle in surgery is inadvisable. Since the recovery of vertebral height is positively correlated with the volume of bone cement injection, it remains unclear whether the risk of this new vertebral fracture is caused by recovery of vertebral height, or secondary effect due to increased volume of bone cement injection^43^. Mu^44^ *et al*. consider that, by comparing with ordinary-viscosity bone cement, vertebroplasty of high-viscosity bone cement features more advantages in spinal function recovery, physiological structure and leakage reduction. How changes in spinal curvature after PKP and recovery of vertebral height correlate with postoperative outcomes requires further research in the future.

The shortcoming of this study is that the method we adopted is a retrospective case study method with less case number and a follow-up period of only one year. The results can be more convincing if randomized controlled trial with a large sample and longer follow-up period are available.

We believe that unilateral puncture for kyphoplasty can effectively treat osteoporotic vertebral compression fractures. The weight of patient, degree of intraoperative cement dispersion and type of injured vertebrae fracture are risk factors affecting its curative effect. Choosing right patient for unilateral transpedicular puncture and improving puncture techniques can improve postoperative patient satisfaction.

## Methods

This is a retrospective case analysis. The experiment was completed in Orthopaedic Department of Hunan Provincial People’s Hospital from March 2016 to March 2017. 70 patients with senile osteoporotic thoracic and lumbar vertebral compression fractures were selected, including 20 males and 50 females, aged 59–82 years old. The interval between injury and surgery was 1–22 days. Vertebrae and number of cases involved in vertebral compression fractures: T6 (1 case), T8 (4 cases), T9 (1 case), T10 (2 cases), T11 (9 cases), T12 (20 cases), L1 (29 cases), L2 (11 cases), L3 (3 cases), L4 (4 cases) and L5 (1 case). Among them, there were 56 patients with single-segment vertebral fractures, 13 patients with two-segment vertebral fractures and 1 patient with three-segment vertebral fractures.

### Inclusion criterion and Exclusion criterion

Patients who were diagnosed with osteoporotic vertebral compression fractures; patients without cardiopulmonary, liver and renal failure or coma; patients without coagulation disorders or bleeding tendency; patients with good corporeity who could tolerate prolonged proneness and surgery. In accordance with the principles of AO (Arbeitsgemeinschaft fur Ostrosynthesefragen, AO Specialty Board for Spine Surgery) classification of spinal fracture, the standard position A1.1 (fractures were only on the upper endplate or the lower endplate) was selected as the patients of fracture type 1, and the standard position A1.2 type (fractures were on both the upper endplate and the lower endplate) was selected as the patients of fracture type 2. Patients without infectious diseases such as vertebral tuberculosis and suppuration on their vertebral bodies; patients without infection around puncture site or on puncture channel.

### Materials

OSTEOPAL V BONE CEMENT (produced by German Heraeus Medical GhbH); Medical Registration No.: GXZJ20143655901, Standard Number of Registered Products: YZB/GER 6661–2014. The product consists of two parts: powder and liquid. The main components of the powder are methyl acrylate-methyl methacrylate polymer, zirconium dioxide, benzoyl peroxide and copper chlorophyll (E141); the main components of the liquid: methyl methacrylate, N, N-dimethyl-p-toluidine, copper chlorophyll (E141) and hydroquinone. The viscosity of this bone cement is very low in the initial stage, so the curing stage is very short. In this way, it is very advantageous for the bone cement to pass through the needle tube. Its physical properties are excellent. Compressive strength ≥87 MPa, elastic modulus ≥3100 MPa, and flexural strength ≥58 MPa. The results of the test indicate that the material has no obvious cytotoxicity and has good blood and tissue compatibility.

### Surgical methods

A patient took prone position after being thoroughly anesthetized. G arm was used for fluoroscopy from normal and lateral positions to locate the injured vertebrae. In surgical area, routine disinfection was carried out and sterile drapes were placed. Under fluoroscopy, the extraneous position on the left side of the injured vertebra at the 10 o’clock direction of the pedicle was used as the needle insertion point, and a 0.4 cm incision was made on skin with a sharp knife; under fluoroscopy, a puncture was performed to insert a cannula with a stylet into the vertebral body through the pedicle; under fluoroscopy, the front end of the cannula was placed at about 0.5 cm from the front of posterior edge of the vertebral body, and then the guide pin and the stylet of the cannula were removed. A drill bit were inserted into the cannula; under fluoroscopy, a channel was drilled in the vertebral body; then the drill bit were pulled out, a balloon was placed, and the pressure was gradually increased to 180 Bmp. It could be seen that the height of the injured vertebra was recovered partially, the balloon expanded evenly and there was no contrast agent leakage. Then, the balloon was taken out, and 4.5 ml of bone cement was slowly and uniformly injected into the vertebral body with a bone cement injector. Under fluoroscopy, it was found that the distribution of bone cement in the vertebral body was relatively uniform and there was no abnormal leakage; in about 15 minutes, the bone cement was solidified; then the cannula was removed. The incision was conventionally bonded and bandaged with a dressing. The operation was very smooth, the patient’s intraoperative and postoperative vital signs were very stable, and there was no postoperative special discomfort in sensation and activity of the lower limbs. The patient could safely return to the ward after being awakened completely from anesthesia.

### Main observation indices

The patients’ general information such as ages, genders, weights, duration of disease, staging of fracture time, number of injured vertebral segments, causes of trauma history, AO classification. VAS scores, vertebral height, Cobb angle of injured vertebra segment, bone cement dispersion, bone cement leakage and complications on the first day before and after the operation and at the time of the last follow-up. The heights of the front, middle and posterior edges of the injured vertebra and the Cobb angle of the responsible vertebral body were measured using SURGIMAP SOFTWARE (NEMARIS Inc.). Each injured vertebra was measured twice by two associate chief physicians or two physicians at above that level, and then the measured results were averaged. On the first day after the surgery and at the last follow-up, X-ray scans were performed to observe the dispersion and leakage of bone cement. The degree of relief of lumbago was assessed by VAS, and the level of the score indicated the severity of the pain.

### Definition of fracture staging

Acute phase (2 weeks), subacute phase (2–8 weeks) and chronic phase (>2 months)^45,46^.

### Assessment of bone cement dispersion

Groups were divided according to the intraoperative or postoperative X-ray images; anteroposterior bone cement projection ≤1/2, lateral bone cement projection ≤1/2, bone cement dispersion ≤25%, and the dispersion level (Grade I). Through the anteroposterior or lateral X-ray images, it was found that the bone cement of either body position ≤1/2, the bone cement of the other body position >1/2, the bone cement dispersion 25–50%, and the dispersion level (Grade II); anteroposterior and lateral bone cement >1/2, bone cement dispersion >50%, and dispersion level (Grade III)^33^.

### Pain score

The pain situation in 3 days and 1 year after surgery was assessed with the VAS method^47^. VAS was represented with a line of 10 cm in length, on which 0 cm indicated no pain and 10 cm indicated the most severe pain. The patients marked their pain points on the line according to their pain perception, and then the distances between the points and the 0 cm point were measured, which were used as quantitative indices of pain values.

### Statistical analysis

The statistical data were processed with SPSS 20.0 software. Postoperative VAS score >5 points was defined as poor clinical efficacy. The relevant factors were used as independent variables, and the clinical efficacy was used as a dependent variable for univariate analysis. Normality test was used for the measurement data, and the measurement data with normal distribution were recorded in the form of $\bar{x}$± s; the comparison between groups was performed by *t* test for independent sample, and the measurement data with non-normal distribution were recorded in the form of Median (P25-P75); the Mann-Whitney test was used for comparison among groups, the count data was recorded in n (%), and the χ2 test was used for comparison among groups. The influencing variables with statistical significance (*p* < 0.05) were screened out, and multivariate analysis was further performed with logistic regression equation. *p* < 0.05 indicated that the difference was statistically significant.

### Ethical Approval and Consent to participate

Informed consent was obtained from all the individual participants included in the study. The study was conducted according to the Helsinki Declaration (Ethical Principles for Medical Research Involving Human Subjects) and was approved by the ethics committee of Hunan Provincial People’s Hospital.

## Data Availability

The datasets used and analyzed during the current study are available from the corresponding author on reasonable request.
